# Empagliflozin protects against renal ischemia/reperfusion injury in mice

**DOI:** 10.1038/s41598-022-24103-x

**Published:** 2022-11-11

**Authors:** Qifeng Wang, Feng Ju, Jiaxue Li, Ting Liu, Yunxia Zuo, Geoffrey W. Abbott, Zhaoyang Hu

**Affiliations:** 1 Laboratory of Anesthesia and Critical Care Medicine, National-Local Joint Engineering Research Centre of Translational Medicine of Anesthesiology, Department of Anesthesiology, West China Hospital, Sichuan University, Chengdu, Sichuan China; 2 Bioelectricity Laboratory, Department of Physiology and Biophysics, School of Medicine, University of California, Irvine, CA USA

**Keywords:** Kidney, Kidney, Acute kidney injury

## Abstract

Renal ischemia/reperfusion (I/R) can induce acute kidney injury. Empagliflozin is a newly developed inhibitor of sodium-glucose cotransporter-2 (SGLT2) approved as an antidiabetic medication for patients with type 2 diabetes mellitus. Despite the established cardioprotective functions of empagliflozin, its protective role in renal I/R is unclear. Here, the present study evaluated the renoprotective effects of empagliflozin in a mouse model of renal I/R injury. Male C57/BL6 mice were allocated to sham-operated, I/R, and empagliflozin groups. Kidney pedicles on both sides were clamped for 45 min and were reperfused for 24 h. Empagliflozin (1 mg/kg) was administered to the mice for 2 days preischemia. The GSK-3β inhibitor SB216763 was administered intravenously at the beginning of reperfusion (0.1 mg/kg). Renal function and histological scores were evaluated. The kidneys were taken for immunohistochemical analysis, western blotting and apoptosis measurements. We found that empagliflozin decreased serum levels of creatinine and urea, reduced the average kidney weight-to-tibia length ratio, attenuated tubular damage, reduced renal proinflammatory cytokine expression and inhibited apoptosis in injured kidneys. Furthermore, empagliflozin increased renal glycogen synthase kinase 3β (GSK-3β) phosphorylation post I/R. Pharmacological inhibition of GSK-3β activity mimicked the renal protective effects offered by empagliflozin. In summary, these results support a protective role of empagliflozin against renal I/R injury.

## Introduction

Renal ischemia/reperfusion (I/R) injury is often seen in kidney transplantation^1^, major vascular surgery^2^, or sepsis^3^. It can induce acute kidney injury (AKI), which is characterized by tubular epithelial cell damage and is associated with significant kidney dysfunction, prolonged hospitalization, subsequent chronic kidney disease development, and high morbidity and mortality rates in patients^4^. Renal I/R injury is currently an incurable perioperative complication; therefore, it is urgent to develop novel strategies to preserve renal function following renal ischemia.

Sodium-glucose cotransporter 2 (SGLT2) inhibitors are new antihyperglycemic drugs that target renal proximal tubules and inhibit glucose reabsorption in patients with type 2 diabetes mellitus (T2DM). Four SGLT2 inhibitors—canagliflozin, dapagliflozin, empagliflozin, and ertugliflozin—are commercially available for clinical use. In addition to their potent blood glucose lowering effect, empagliflozin and canagliflozin have been shown to exert significant renal protective effects in T2DM patients^5,6^. In addition, the recent CREDENCE trial further showed that continued treatment with canagliflozin was effective at improving renal function in patients with type 2 diabetes with chronic kidney disease (CKD)^7^. Importantly, these beneficial renal effects have extended to nondiabetic patients. For example, the DAPA-CKD trial showed that dapagliflozin was effective at reducing the risk of CKD progression or death from renal causes in participants with CKD with or without T2DM when compared with those in the placebo group^8^. However, regarding renal I/R injury in nondiabetic individuals, the current understanding of SGLT2 inhibitor-induced renoprotection in the perioperative period is limited. Scattered reports indicate that dapagliflozin ameliorates acute renal tubule injury and improves renal function in a murine model of acute kidney injury^9^, while luseogliflozin prevents renal fibrosis after prolonged renal I/R injury in mice^10^.

We recently showed that empagliflozin was capable of reducing reperfusion-induced arrhythmogenesis in healthy rats after myocardial I/R injury^11^ or ameliorated lung damage after pulmonary I/R injury^12^. However, it remains unclear whether empagliflozin has renoprotective effects against renal I/R injury in a glucose-independent manner.

Meanwhile, understanding the underlying mechanisms of renoprotection may provide avenues for novel therapeutic strategies for urologists and anesthetists to manage perioperative renal injury and reduce renal deterioration after renal ischemia. GSK-3β is a serine/threonine protein kinase and an essential regulator of cellular functions. It is a major downstream molecule of the reperfusion injury salvage kinase (RISK) pathway^13^. Abundant evidence has indicated that GSK-3β phosphorylation (via inhibition of GSK-3β activity) could exert a protective effect against I/R injury in hearts^14^, brains^15^ and livers^16^. GSK-3β can be phosphorylated on Ser9 by various protective stimuli, such as volatile anesthetics^17^ and ischemic conditioning^14^.

Thus, we hypothesize that empagliflozin protects the kidney against I/R injury similar to GSK-3β inhibition.

Therefore, the aims of the current study were to test the hypothesis that empagliflozin is renoprotective in an in vivo nondiabetic mouse model of renal I/R injury. Furthermore, we aimed to elucidate the underlying molecular mechanism responsible for any observed empagliflozin-mediated renoprotection.

## Materials and methods

### Animals

This study was carried out in compliance with the ARRIVE (Animal Research: Reporting of In Vivo Animal Experiments) guidelines. Our research and protocols were approved by the Institutional Animal Care and Use Committee of Sichuan University (Chengdu, Sichuan, China) (Approval No: 20211201 A) and were in compliance with the principles for the care and use of laboratory animals (Guide, Eighth edition, 2011). C57/BL6 male mice (weighing 20 to 30 g, average age 10 months) (Chengdu Dashuo Experimental Animal Co., Ltd., Chengdu, China) were utilized in this study. Mice were housed in a specific-pathogen free animal facility with a circadian rhythm of 12 h light/12 h darkness. All mice had free access to water and food.

### Study design

**Figure 1:**
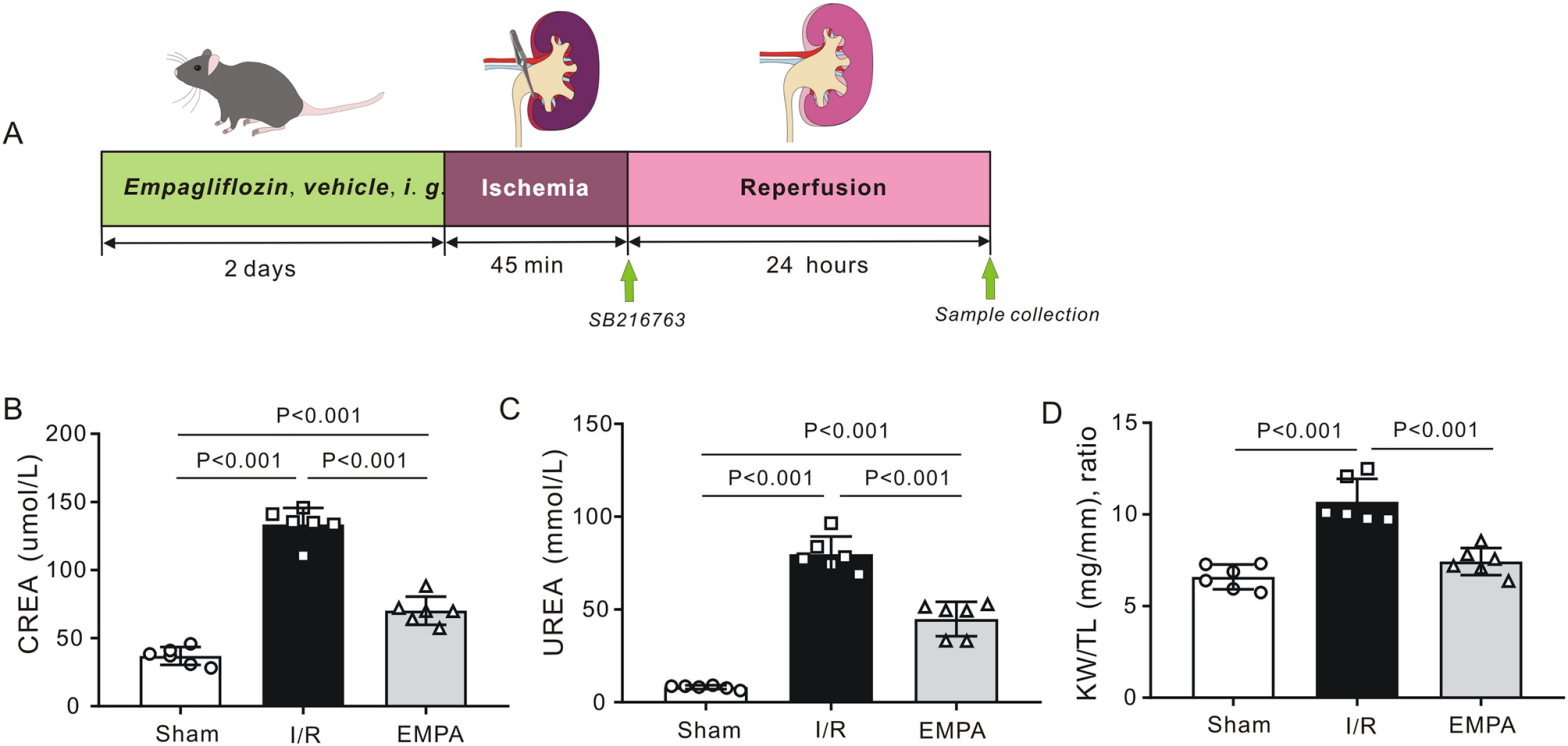
Empagliflozin alleviates kidney damage after renal I/R injury in mice. (**A**) A schematic representation of the experimental design. (**B**, **C**) Serum levels of creatinine (CREA) (**B**) and blood urea (UREA) (**C**) in mice subjected to 45 min of renal ischemia followed by 24 h of reperfusion. n = 6 per group. *Sham* sham-operated, *I/R* ischemia/reperfusion, *EMPA* empagliflozin. (**D**) Kidney weight-to-tibia length (KW/TL) ratio of the mice treated with empagliflozin or vehicle post-IR. n = 6 per group. Data shown are the mean ± SD. Significant differences between groups were determined by one-way ANOVA (**B**–**D**).

Mice were randomly divided into 5 groups: (1) sham-operated group (Sham, n = 6) received physiological saline (0.9% NaCl) solution; (2) ischemia/reperfusion group (I/R, n = 6) received physiological saline (0.9% NaCl) solution; (3) empagliflozin group (EMPA, n = 6) received empagliflozin (1 mg/kg, Ingelheim am Rhein, Germany); (4) I/R + inhibitor group (SB + I/R, n = 6); and (5) empagliflozin + inhibitor group (SB + EMPA, n = 6). We evaluated the protective effect of EMPA in the early phase of renal I/R. After anesthesia with phenobarbital sodium (50 mg/kg, i.p.), mice were kept in a fixed supine position on a heating pad. A longitudinal abdominal median incision was performed to expose the kidneys. Kidney pedicles on both sides were exposed and isolated. The renal pedicles in sham-operated rats were isolated without occlusion. The surgical procedure used in the current study is based on previous publications^18,19^. The renal pedicles were clamped with nontraumatic microvascular clamps in the I/R and EMPA groups for 45 min to induce renal ischemia. After that, clamps were removed to permit reperfusion for 24 h before tissue collection. Abdominal median incisions were closed with 5–0 silk sutures (Ethicon, Inc., Somerville, NJ, USA) and the mice were replaced in their previous environment after the surgery. A heating pad of 37 °C was used throughout the experiment to maintain body temperature. Nalbuphine (2 mg/kg, S.C.) was used for analgesia after renal surgery. An equal amount of vehicle (saline and empagliflozin) was given twice to all animals orally. The first dose was given 24 h before renal ischemia. The second dose was given 1 h before renal ischemia. SB216763, a specific GSK-3β inhibitor (0.1 mg/kg, MedChemExpress, Monmouth Junction, NJ, USA), was intravenously administered to the mice in the I/R and EMPA groups from the femoral vein at the beginning of reperfusion^14^ (Fig. 1A).

### Sample collection

After 24 h of reperfusion, animals were sacrificed with an overdose of pentobarbital sodium (200 mg/kg, i.p.). Death was confirmed by termination of respiration and cardiac activity. Then, blood was taken from the heart and centrifuged (1000×*g*, 10 min, 4 °C). The supernatant was taken for biochemical analysis. Kidneys were then taken, weighed and stored in a − 80 °C freezer for subsequent western blot analysis or fixed with 4% paraformaldehyde for pathological analysis. Tibia length was measured using a Vernier caliper.

### Biochemical analysis

For determination of renal function, serum concentrations of creatinine (CREA) and urea (UREA) were measured using a Mindray BS-120 Chemistry Analyzer (Mindray Medical, Shenzhen, China).

### Histological staining

Fixed kidneys were embedded with paraffin wax and cut into 4-μm-thick sections. Then, the kidneys were deparaffinized, rehydrated and stained with hematoxylin and eosin (H&E) or Alcian blue-periodic acid-Schiff (AB-PAS) (Solarbio, Beijing, China). Histological assessment was performed by two independent researchers in a blinded manner according to an established grading scale of 0–4^20^ as follows: 0: no injury, (1) the injured area was less than 25% of the tissue in the field of view, (2) the injured area was between 25 and 50%, (3) the injured area was between 50 and 75%, (4) the injured area was more than 75%. Renal damage is characterized by tubular dilation or swelling, necrosis, brush border loss interstitial infiltration and intratubular cast formation. At least 10 randomly selected fields of the cortical tissues were examined for each section.

### TUNEL assay

Apoptotic cells were evaluated by terminal deoxynucleotidyl transferase (TdT) dUTP nick-end labeling (TUNEL) assay in cortical tissues via an In Situ Cell Death Detection Kit (DeadEnd™ Fluorometric TUNEL system, Promega Corporation, Madison, WI, USA) in accordance with the manufacturer's instructions. The average number of TUNEL-positive cells, calculated as a percentage of the total nuclei population, was counted from 10 randomly selected fields under a Nikon fluorescence microscope (Eclipse Ni-E, Nikon, Tokyo, Japan).

### Western blot analysis

Renal cortices were washed with ddH_2_O and homogenized in ice-cold RIPA buffer containing 50 mM Tris–HCl (pH 7.4), 150 mM NaCl, 1% NP-40, 1 mM EDTA, 0.25% sodium deoxycholate, phosphatase inhibitor cocktail, and a protease inhibitor cocktail (Sigma Chemical Co., St. Louis, MO, USA). The tissues were ground with a manual grinder on ice (Fisher Scientific, Hampton, NH, USA), followed by centrifugation for 10 min at 4 °C at 10,000×*g*. The protein concentration was measured and calculated using the BCA method (Pierce, Rockford, IL USA). The proteins were then resolved on a 12% Tris–glycine SDS-PAGE gel and transferred to a nitrocellulose blotting membrane (Pall Corporation, Pensacola, FL, USA). The membranes were blocked with 5% nonfat milk dissolved in PBST (PBS containing 0.1% Tween 20). Primary antibodies included IL-6, TNF-α (all 1:1000, Affinity Biosciences, Cincinnati, OH, USA), GAPDH (1:2000, YEASEN Bio. Inc., Shanghai, China), phosphorylated GSK-3β (p-GSK-3β) (Ser9), total GSK-3β (t-GSK-3β), phosphorylated ERK1/2 (p-ERK1/2) (Thr202/Tyr204), total ERK1/2 (t-ERK), phosphorylated STAT-3 (Tyr705)(p-STAT3), total STAT-3 (t-STAT3), phosphorylated STAT-5 (Tyr694) (p-STAT5), and total STAT-5 (t-STAT5) (all 1:1000, Cell Signaling, Danvers, MA, USA). After incubation overnight, peroxidase-conjugated goat anti-rabbit secondary antibody (1:5000, Bio-Rad, Hercules, CA, USA) was used. A super ECL detection reagent (YEASEN Bio. Inc., Shanghai, China) was utilized to detect immunoblotted bands. ImageJ (National Institutes of Health, Bethesda, MD, USA) was applied for densitometric analysis.

### Immunohistochemical analysis

After fixation with 4% paraformaldehyde, kidney tissues were embedded with paraffin wax and cut into 4-μm-thick sections. Next, they were deparaffinized and rehydrated. Antigen was retrieved with citrate buffer solution (pH 6.0) according to the recommendation from antibody manuals. Slices were heated in a microwave for 8 min. Next, a hydrogen peroxide blocking reagent (Abcam, Waltham, MA, USA) was used for 30 min at room temperature to block endogenous peroxidase. Tissues were then incubated with 10% goat serum for 40 min at 37 °C before interaction with specific antibodies. Primary antibodies against IL-6, TNF-α, NGAL, Bcl-2, Bax, β-catenin and Nrf2(all 1:200, Affinity Biosciences, Cincinnati, OH, USA) and phosphorylated GSK-3β (p-GSK-3β, 1:200, Cell Signaling, Danvers, MA, USA) were used to incubate slices at 4 °C overnight. Then, peroxidase-conjugated goat anti-rabbit secondary antibody (Santa Cruz Biotechnology, Santa Cruz, CA) was diluted to a concentration of 1:250 with phosphate-buffered saline for incubation of renal slices for 30 min at 37 °C. The sections were then incubated with 3,3-diaminobenzidine (DAB, Beijing Zhongshan Golden Bridge Biotechnology, Beijing, China). Images were taken under an inverted microscope (CAST system, Olympus A/S, Ballerup, Denmark). Ten fields of each slide were chosen at random. Image-Pro Plus software (Media Cybernetics Inc., Carlsbad, CA, USA) was applied to analyze the mean intensity of the positively stained area.

### Statistical analysis

Data are expressed as the mean ± standard deviation (SD). Statistical analysis was performed using GraphPad Prism 8 (GraphPad Software, Inc., La Jolla, CA, USA) software. Data were analyzed with one-way analysis of variance (ANOVA). The Levene’s test was used to examine the equality of variances. Between-group comparisons were performed using the Student–Newman–Keuls test if the variance was equal; if not, Dunnett’s T3 test was applied. P < 0.05 was regarded as statistically significant.

## Results

### Empagliflozin reduces renal damage post I/R

**Figure 2:**
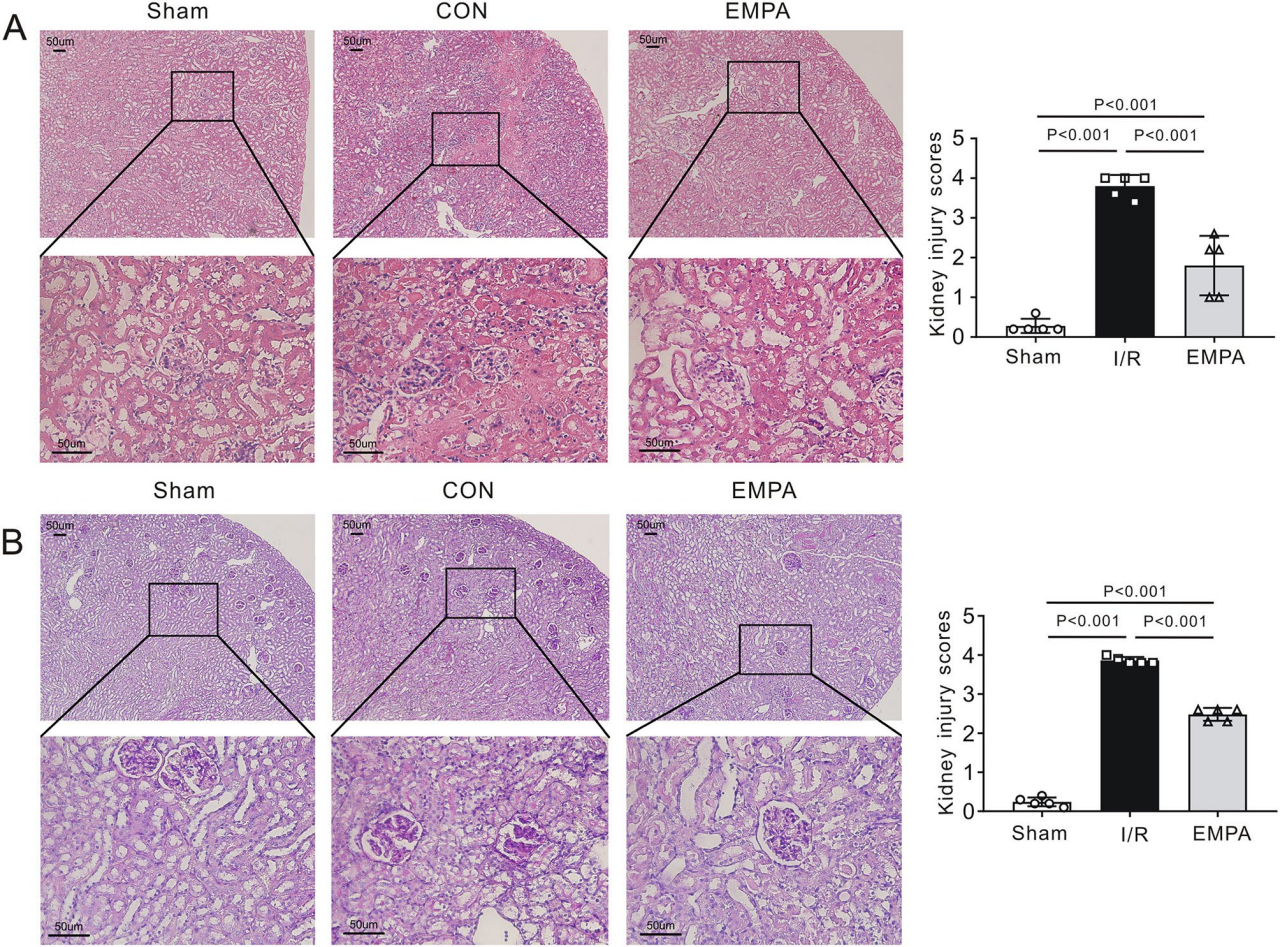
The effect of empagliflozin on renal histological changes after I/R injury. (**A**) *Left*, Representative images of kidney sections stained with H&E after renal I/R injury. n = 5 mice per group. *Sham* sham-operated, *I/R* ischemia/reperfusion, *EMPA* empagliflozin. Scale bars, 50 μm. *Right panel*, Differences between the histopathological scores of groups (n = 5 mice per group). (**B**) *Left*, Representative kidney sections stained with PAS after renal I/R injury. Scale bars, 50 μm. *Right panel*, renal tubular injury scores in each group. n = 5 per group. Data shown are the mean ± SD. Significant differences between groups were determined by one-way ANOVA (**A**, **B**).

The impact of empagliflozin on kidney I/R injury is not known. We therefore tested serum levels of creatinine (CREA) and urea (UREA), the most widely accepted parameters for evaluating kidney function. We found that serum levels of CREA (133.5 ± 12.2 μmol/L, *P* < 0.001 vs. Sham) and UREA (79.8 ± 9.5 mmol/L, *P* < 0.001 vs. Sham) were markedly increased following renal I/R in I/R mice, indicating that I/R caused renal injury. However, the values of CREA (70.2 ± 10.3 μmol/L, *P* < 0.001 vs. I/R) and UREA (28.3 ± 5.7 mmol/L, *P* < 0.001 vs. I/R) in EMPA mice were significantly lower than those of mice in the I/R group (Fig. 1B,C). We also measured the average kidney weight-to-tibia length (KW/TL) ratio to quantify kidney swelling. As expected, mice treated with empagliflozin (7.4 ± 0.7, P < 0.001 vs. I/R) had a much lower KW/TL ratio than did those receiving vehicle alone (10.7 ± 1.2, Fig. 1D). I/R kidneys demonstrated significant tubular injury with evidence of renal swelling, tubular vacuolation, dilatation, protein cast formation and necrosis. Using H&E staining, we quantified renal pathological changes post I/R and found that the degree of renal injury was less severe in empagliflozin-treated mice (1.8 ± 0.7) than I/R mice (3.8 ± 0.3, P < 0.001, Fig. 2A). Consistent with this finding, empagliflozin administration ameliorated tubular structural abnormalities as observed by PAS staining (P < 0.001 vs. I/R, Fig. 2B).

### Empagliflozin reduces renal proinflammatory cytokine expression

**Figure 3:**
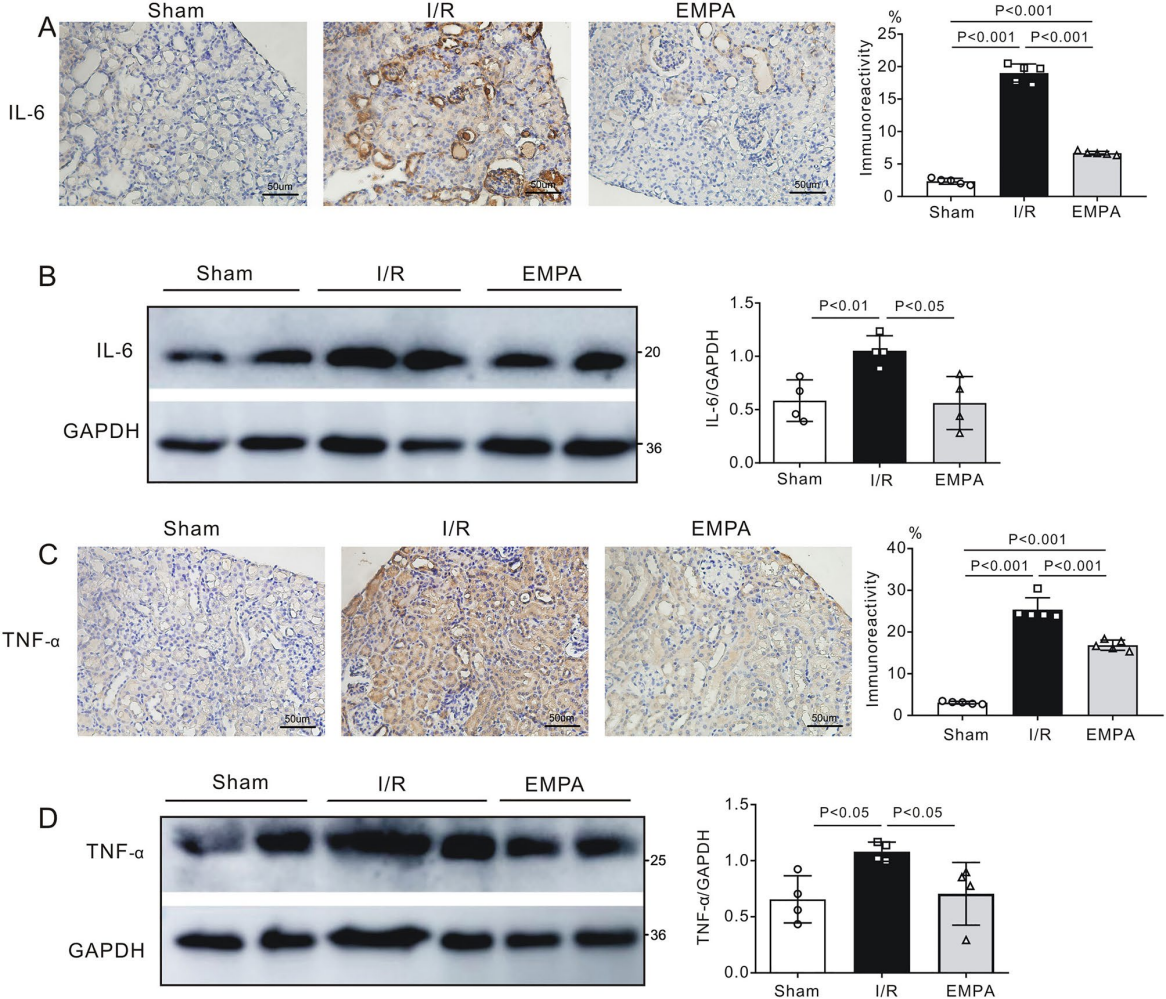
Empagliflozin alters inflammatory responses after renal I/R injury. (**A**) *Left,* Representative images of immunostaining for interleukin-6 (IL-6) in kidney sections after renal I/R injury. Scale bars, 50 μm. *Sham* sham-operated, *I/R* ischemia/reperfusion, *EMPA* empagliflozin. *Right,* the level of immunoreactivity of IL-6 in mouse kidneys post I/R. Each group, n = 5. (**B**) *Left,* Representative blots for IL-6 and GAPDH in mouse kidneys; *Right,* IL-6/GAPDH ratio after renal I/R injury. n = 4 per group. (**C**) *Left,* Representative images of immunostaining for tumor necrosis factor-α (TNF-α) in kidney sections after renal I/R injury. Scale bars, 50 μm. *Sham* sham-operated, *I/R* ischemia/reperfusion, *EMPA* empagliflozin. *Right panel,* TNF-α immunoreactivity levels in mouse kidneys post I/R. Each group, n = 5. (**D**) *Left,* Representative blots for TNF-α and GAPDH in mouse kidneys; *Right,* TNF-α/GAPDH ratio after renal I/R injury. n = 4 per group. Data shown are the mean ± SD. Significant differences between groups were determined by one-way ANOVA (**A**–**D**).

Renal expression of the proinflammatory cytokines interleukin-6 and tumor necrosis factor-α was determined using immunohistochemistry and western blotting**.** As shown in Fig. 3A, IL-6 immunoreactivity was detected in each group. The expression of IL-6 was significantly elevated in vehicle-treated, I/R mouse kidneys (I/R, P < 0.001 vs. Sham). However, administration of empagliflozin markedly reduced the positive expression of IL-6 protein within the glomeruli and tubules of I/R kidneys (EMPA, P < 0.001 vs. I/R). Consistent with our immunohistochemistry findings, western blot results showed that the ratio of IL-6 to GAPDH was approximately halved in empagliflozin-treated I/R (EMPA) kidneys when compared with vehicle-treated I/R kidneys (P < 0.05), suggesting that empagliflozin was capable of altering inflammatory responses post-I/R (Fig. 3B). We also quantified the expression levels of TNF-α. We found that vehicle-treated (I/R) mice showed a greater increase in renal TNF-α expression following I/R than sham mice (P < 0.001 or P < 0.05). However, treatment with empagliflozin significantly attenuated renal TNF-α expression (P < 0.001 or P < 0.05 vs. I/R, Fig. 3C,D).

### Empagliflozin decreases the expression of NGAL in mouse kidney post I/R

**Figure 4:**
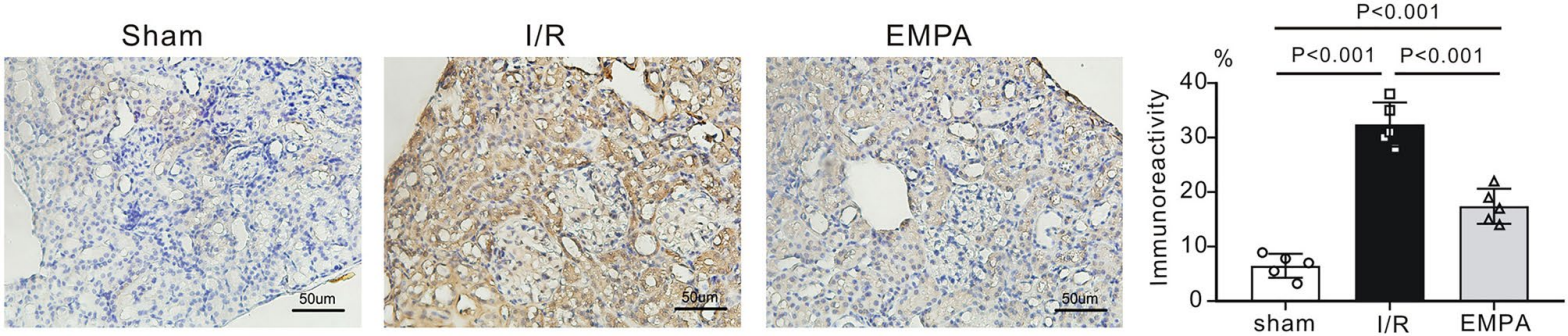
The effect of empagliflozin on renal NGAL expression levels after renal I/R injury. *Left,* Representative images of immunostaining for neutrophil gelatinase-associated lipocalin (NGAL) in kidney sections after renal I/R injury. Scale bars, 50 μm. *Sham* sham-operated, *I/R* ischemia/reperfusion, *EMPA* empagliflozin. *Right panel,* NGAL immunoreactivity levels in mouse kidneys post I/R. n = 5 per group. Data shown are the mean ± SD. Significant differences between groups were determined by one-way ANOVA.

Neutrophil gelatinase-associated lipocalin (NGAL) serves as a tubule injury marker in acute kidney injury^21^. To evaluate the efficiency of empagliflozin in response to renal I/R injury, we examined the renal expression levels of NGAL by immunohistochemistry**.** We showed that NGAL expression was abundant in I/R kidneys following I/R when compared with sham kidneys (P < 0.001), but the expression was decreased in the kidneys treated with empagliflozin after I/R injury (P < 0.001 vs. I/R, Fig. 4).

### Empagliflozin inhibits renal apoptosis post I/R

**Figure 5:**
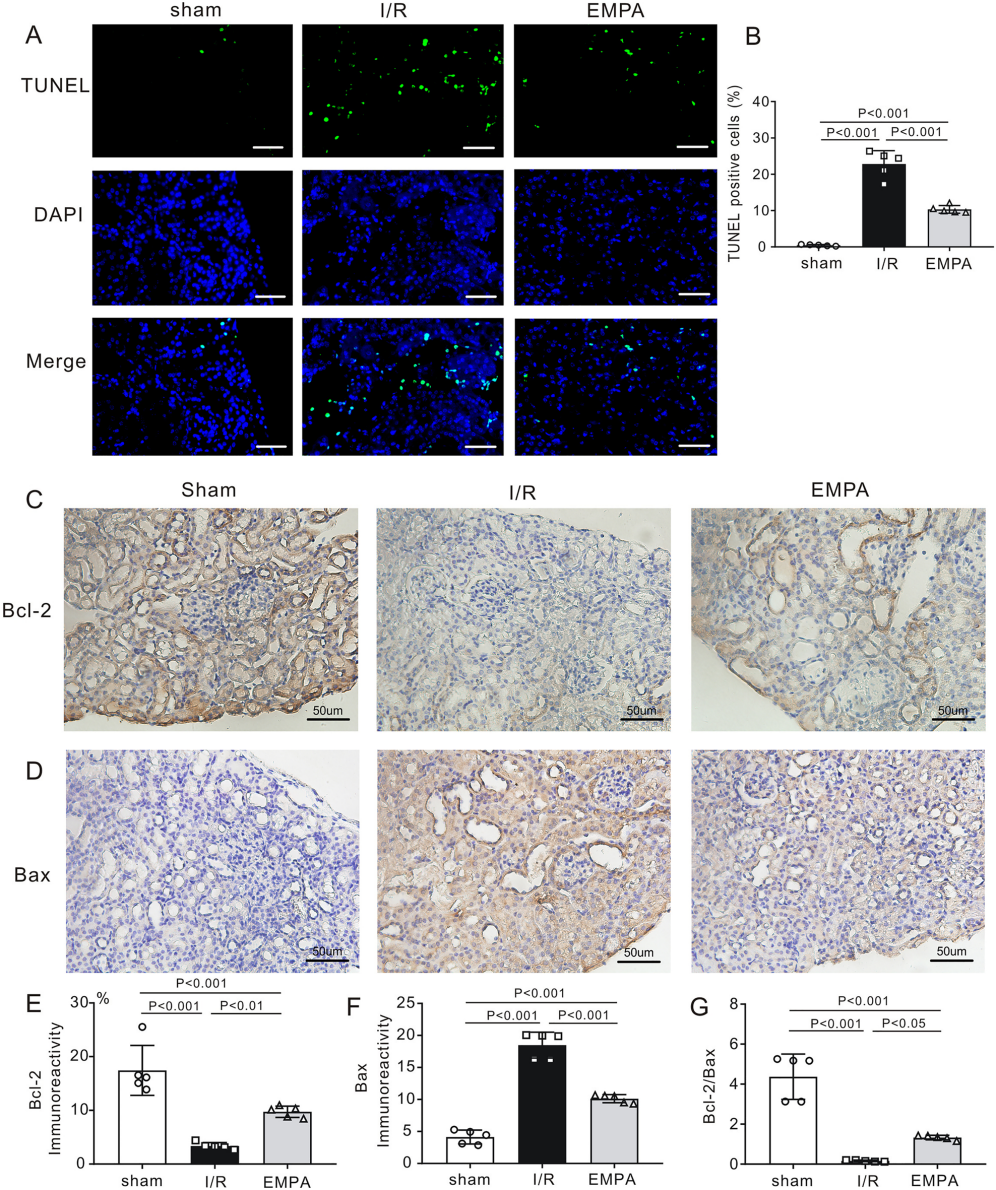
Empagliflozin inhibits renal apoptosis post-I/R. (**A**) *Left,* Representative images of TUNEL staining in mouse kidney sections. Scale bars, 50 μm. *Sham* sham-operated, *I/R* ischemia/reperfusion, *EMPA* empagliflozin. TUNEL-positive cells were stained green, and nuclei were stained with DAPI (blue). (**B**) The ratio of TUNEL-positive cells. n = 5 per group. (**C**, **D**) Representative images of immunostaining for Bcl-2 (**C**) and Bax (**D**) in kidney sections after renal I/R injury. Each group, n = 5. Scale bars, 50 μm. (**E–G**) The analysis of Bcl-2 (**E**), Bax (**F**) and Bcl-2/Bax (**G**) protein expression in mouse kidneys post-I/R. Each group, n = 5. Data shown are the mean ± SD. Significant differences between groups were determined by one-way ANOVA (**B**, **E**–**G**).

To examine the renoprotective effect of empagliflozin on apoptosis, TUNEL staining was applied to kidney sections. As shown in Fig. 5A and B compared to sham mice (0.5 ± 0.2%), renal I/R injury caused massive renal apoptosis in I/R mice (22.8 ± 3.7%, P < 0.001). However, empagliflozin effectively reduced the ratio of apoptotic cells (EMPA, 10.3 ± 1.1%, P < 0.001 vs. I/R). Furthermore, we also examined the expression levels of apoptosis-related proteins. Bcl-2 (anti-apoptotic protein) and Bax (pro-apoptotic protein) immunoreactivities were detected in the kidneys in each experimental group (Fig. 5C,D). Importantly, empagliflozin effectively increased the renal expression of Bcl-2 protein (*P* < 0.01, vs. I/R, Fig. 5E) and reduced Bax expression (*P* < 0.001, vs. I/R, Fig. 5F), resulting in a greater Bcl-2-to-Bax ratio (*P* < 0.05, vs. I/R, Fig. 5G).

### Empagliflozin alters GSK-3β phosphorylation following renal I/R injury

**Figure 6:**
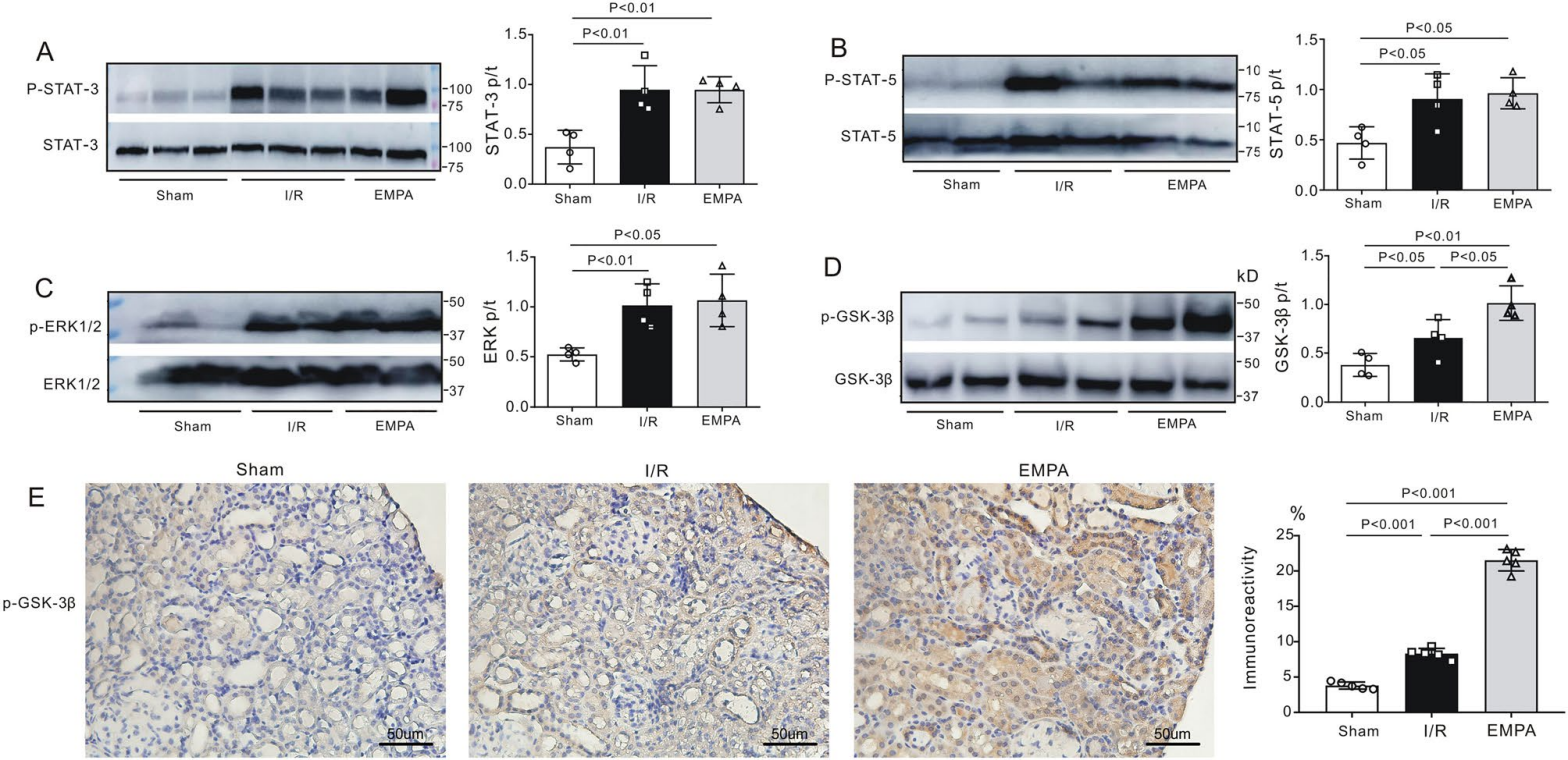
The effect of empagliflozin on renal GSK-3β phosphorylation post-I/R. (**A–D**) *Left,* Representative western blots of renal p-STAT-3 and t-STAT-3 (**A**), p-STAT-5 and t-STAT-5 (**B**), p-ERK1/2 and t-ERK1/2 (**C**), and p-GSK-3β and t-GSK-3β (**D**) after I/R injury. *Sham* sham-operated, *I/R* ischemia/reperfusion, *EMPA* empagliflozin. *Right, Relative* abundance of protein phosphorylation levels was normalized to total protein. n = 4 per group. (**E**)* Left,* Representative images of immunostaining for p-GSK-3β in kidney sections after renal I/R injury. Scale bars, 50 μm. *Right,* p-GSK-3β immunoreactivity levels. Each group, n = 5. Data shown are the mean ± SD. Significant differences between groups were determined by one-way ANOVA (**A**–**E**).

It has been shown that empagliflozin may protect the heart via activation of multiple signaling pathways related to cell survival^11^. To further explain its renoprotective effects, we defined the roles of several vital signaling molecules associated with I/R injury, such as STAT-3, STAT-5, ERK1/2 and GSK-3β. We found that the phosphorylation levels of STAT-3 (Fig. 6A), STAT-5 (Fig. 6B) and ERK1/2 (Fig. 6C) were increased in I/R kidneys after renal I/R compared with sham kidneys (P < 0.05 or P < 0.01). However, empagliflozin treatment did not further enhance their phosphorylation levels (P > 0.05 vs. I/R). Notably, low levels of phospho-GSK-3β expression were detected in the sham group (0.4 ± 0.1), while a significant phospho-GSK-3β-positive signal was detected in I/R-treated and vehicle-treated I/R kidneys (0.7 ± 0.2, P < 0.05 vs Sham). Interestingly, the phosphorylation level of GSK-3β was more than 1.4-fold greater in the empagliflozin-treated kidneys (1.0 ± 0.2) than in the I/R kidneys after I/R injury (P < 0.05, Fig. 6D). Meanwhile, phosphorylated GSK-3β immunoreactivity was also detected in the kidney sections using immunohistochemistry. Consistent with our western blot results, the expression of phospho-GSK-3β was increased in the I/R mice after I/R (P < 0.001 vs, Sham); however, the phospho-GSK-3β signal in the empagliflozin-treated kidneys **(**21.5 ± 1.5) was significantly higher than that in the vehicle-treated I/R kidneys (8.3 ± 0.7, P < 0.001, Fig. 6E).

### Inhibition of GSK-3β activity mimics the renoprotective effect of empagliflozin

**Figure 7:**
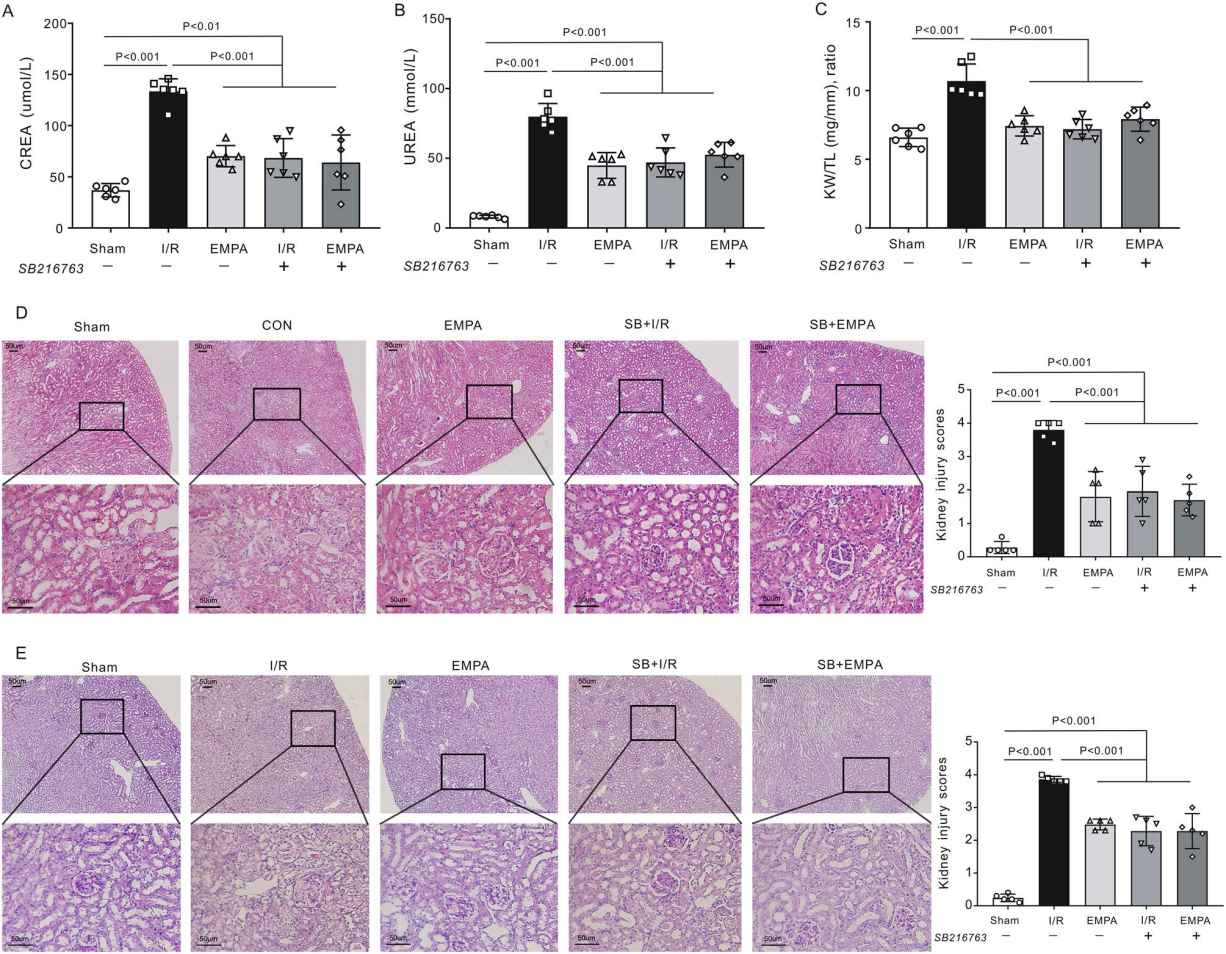
Pharmacological inhibition of GSK-3β mimics empagliflozin-stimulated renal protection. (**A**, **B**) Serum levels of creatinine (CREA) (**A**) and urea (UREA), (**B**) in mice with (+) or without (−) SB216763 treatment. n = 6 per group. *Sham* sham-operated, *I/R* ischemia/reperfusion, *EMPA* empagliflozin. Values for sham, I/R and empagliflozin mice are repeated from Fig. 1B and C for comparison. (**A**) Kidney weight-to-tibia length (KW/TL) ratio of the mice with (+) or without (−) SB216763. n = 6 per group. Values for sham, I/R and empagliflozin mice are repeated from Fig. 1D for comparison. (**D–E**) *Left,* Representative images of kidney sections stained with H&E (**D**) or PAS (**E**) in the presence (+) or absence (−) of SB216763 after renal I/R. n = 5 mice per group. Sham: sham-operated, I/R: ischemia/reperfusion. *EMPA* empagliflozin. Scale bars, 50 μm. *Right panel*, renal damage scores in each group. Values for sham, I/R and empagliflozin mice are repeated from Fig. 2A and B for comparison. Data shown are the mean ± SD. Significant differences between groups were determined by one-way ANOVA (**A**–**E**).

**Figure 8:**
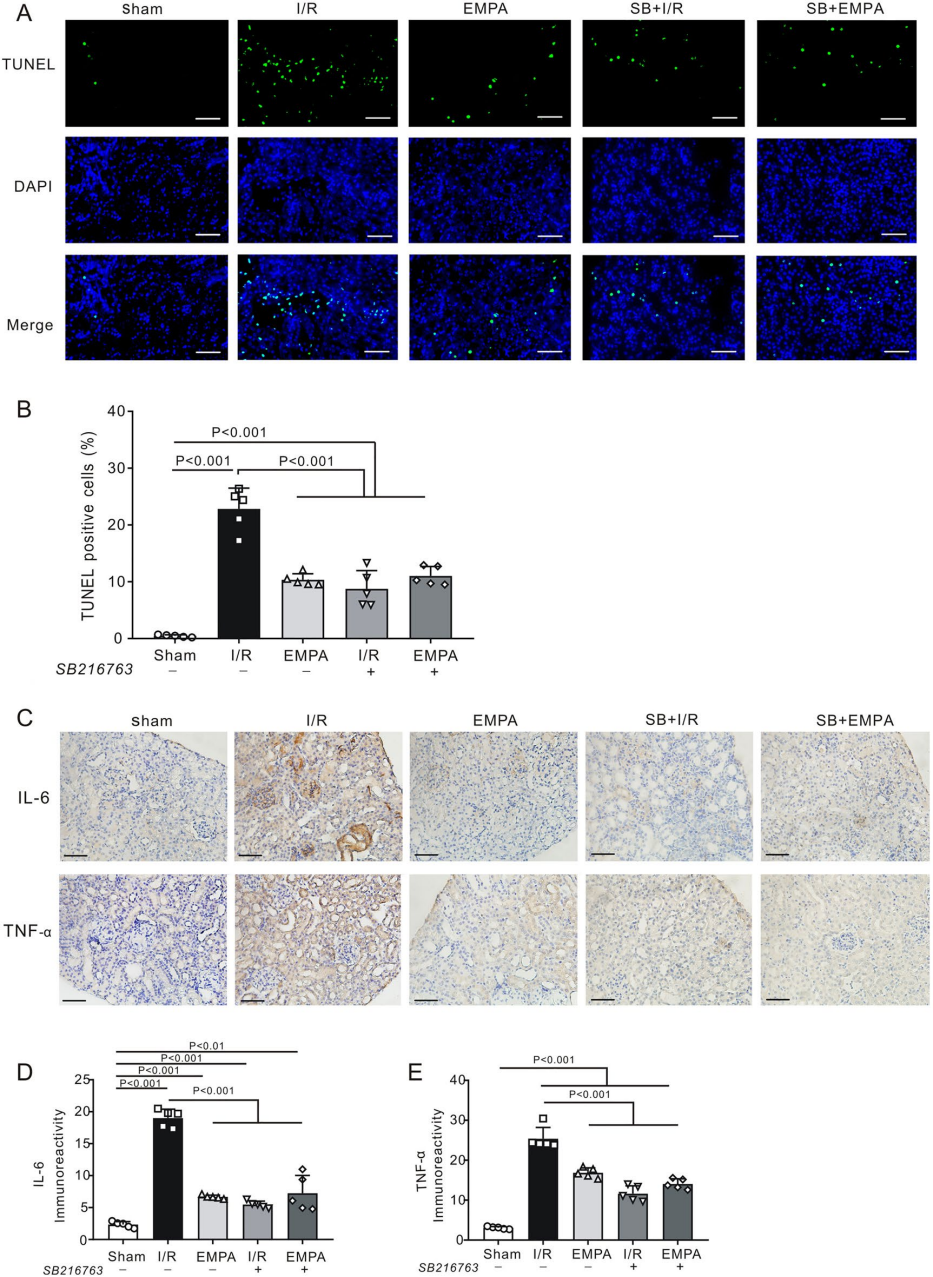
SB216763 inhibits renal apoptosis and the inflammatory response after I/R injury. (**A**) Representative images of renal apoptosis in the presence (+) or absence (–) of SB216763 after renal I/R. *Sham* sham-operated, *I/R* ischemia/reperfusion, *EMPA* empagliflozin. Scale bars, 50 μm. (**B**) The ratio of TUNEL-positive cells. n = 5 per group. Values for sham, I/R and empagliflozin mice are repeated from Fig. 5B for comparison. Data shown are the mean ± SD. Significant differences between groups were determined by one-way ANOVA. (**C**) Representative images of immunostaining for IL-6 and TNF-α in kidney sections after renal I/R injury. Scale bars, 50 μm. Sham: sham-operated, I/R: ischemia/reperfusion, *EMPA*: empagliflozin. (**D**) IL-6 immunoreactivity levels in mouse kidneys post I/R. Each group, n = 5. Values for sham, I/R and empagliflozin mice are repeated from Fig. 3A for comparison. (**E**) TNF-α immunoreactivity levels in mouse kidneys post I/R. Each group, n = 5. Values for sham, I/R and empagliflozin mice are repeated from Fig. 3C for comparison.

**Figure 9:**
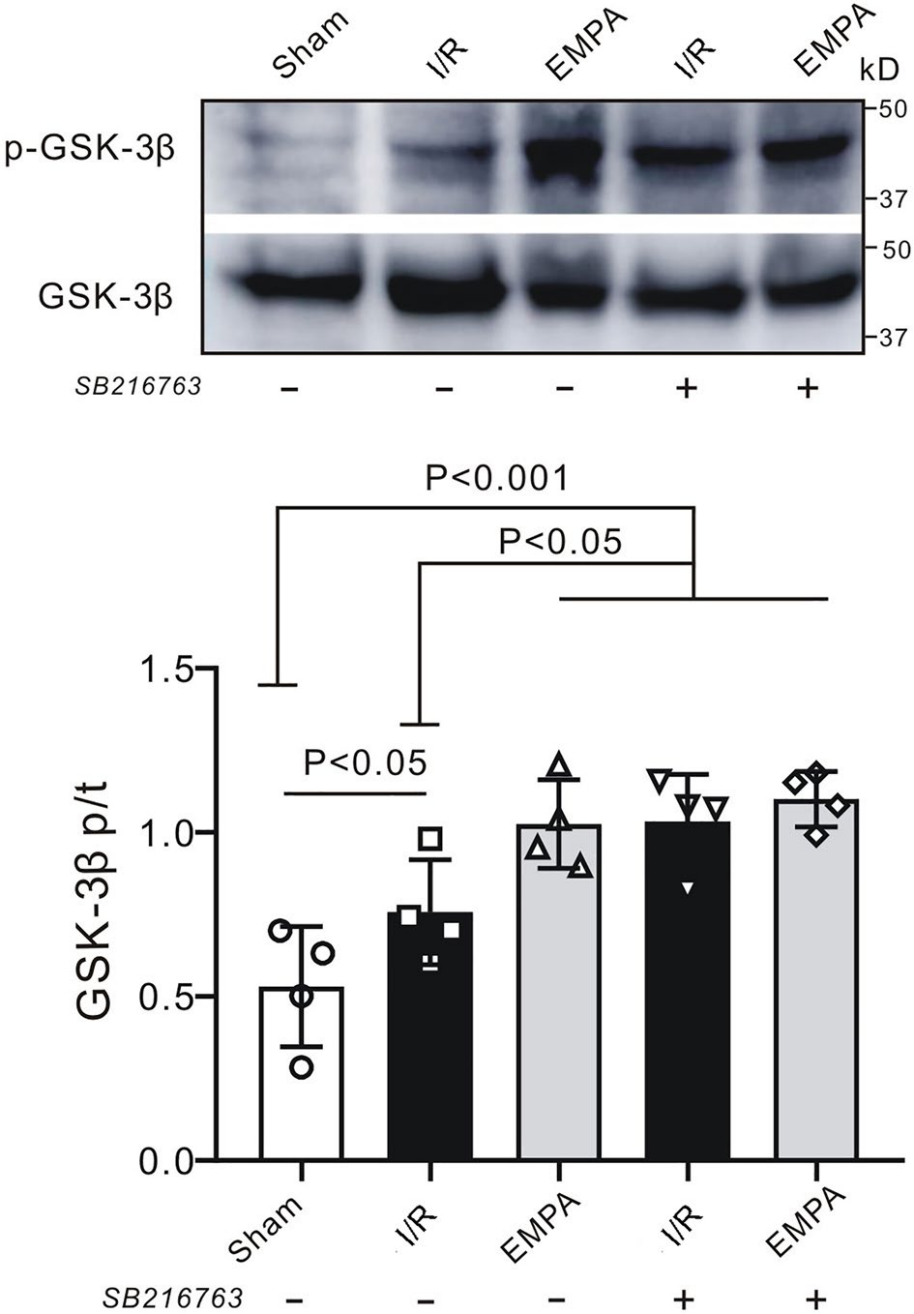
GSK-3β phosphorylation after SB216763 treatment. (**A**) Representative western blot of renal p-GSK-3β and t-GSK-3β after I/R injury. *Sham* sham-operated, *I/R* ischemia/reperfusion, *EMPA* empagliflozin. (**B**) p-GSK-3β/t-GSK-3β ratio. n = 4 per group. Data shown are the mean ± SD. Significant differences between groups were determined by one-way ANOVA.

**Figure 10:**
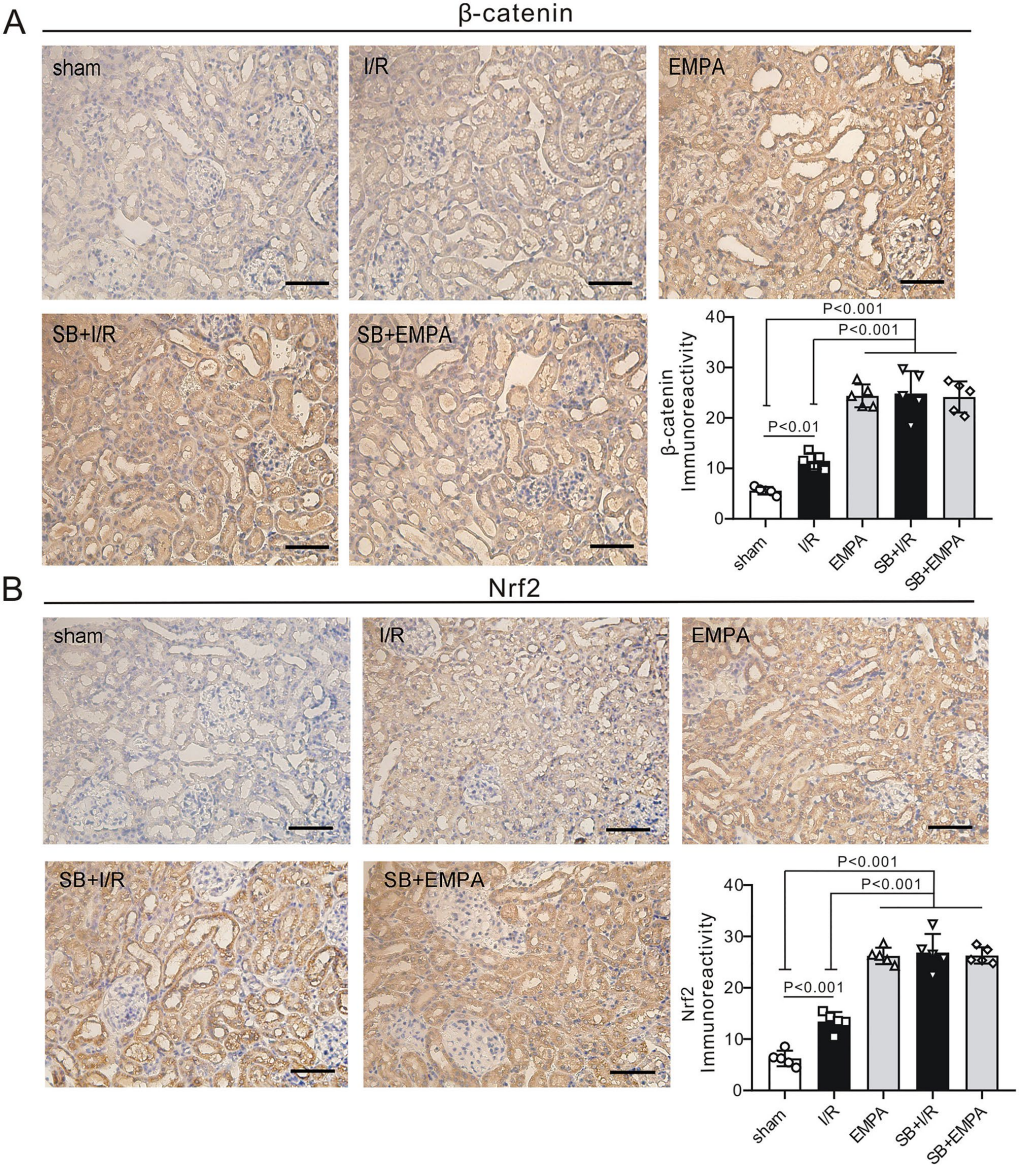
The effect of GSK-3β inhibition on GSK-3β downstream targets. Upper left: Representative images of immunostaining for β-catenin (**A**) and Nrf2 (**B**) in kidney sections after renal I/R injury. Scale bars, 50 μm. *Sham* sham-operated, *I/R* ischemia/reperfusion, *EMPA* empagliflozin, *SB* SB216763. Lower right: immunoreactivity levels of β-catenin and Nrf2 in mouse kidneys post I/R. Each group, n = 5.

We found that empagliflozin enhanced GSK-3β phosphorylation following renal I/R. It has also been suggested that inhibition of GSK-3β activity (via phosphorylation) limits cardiac infarction^14^. To further confirm the role of GSK-3β in renoprotection, we applied a specific GSK-3β inhibitor, SB216763, at the onset of renal reperfusion in our study. We observed that inhibition of GSK-3β exerted a therapeutic effect similar to empagliflozin. For example, the serum concentrations of CREA (68.4 ± 18.9 μmol/L, P < 0.001 vs. I/R, Fig. 7A) and UREA (47.1 ± 10.4 mmol/L, P < 0.001 vs. I/R, Fig. 7B) were reduced in SB216763-treated I/R kidneys to levels similar to those in the empagliflozin-treated group in the absence of SB216763 (CREA:70.2 ± 10.3 μmol/L, UREA: 44.9 ± 9.2 mmol/L). Moreover, I/R group mice treated with SB216763 exhibited a 1.5-fold lower KW/TL ratio than noninhibitor-treated I/R mice post I/R (P < 0.001, Fig. 7C). H&E staining results confirmed that the degree of renal injury was significantly attenuated in SB216763-treated I/R mice (2.0 ± 0.7, P < 0.001 vs I/R: 3.8 ± 0.3) to levels seen in empagliflozin-treated I/R mice (1.8 ± 0.7, Fig. 7D). In accordance with these data, histological observation of PAS staining on mouse kidney sections also revealed that those who received SB216763 in addition to vehicle had a significantly lower renal tubular injury score (2.3 ± 0.4) than did those receiving vehicle alone post I/R (3.9 ± 0.1, P < 0.001, Fig. 7E). Consistently, SB216763-treated I/R kidneys exhibited reduced numbers of renal apoptotic nuclei when compared with I/R kidneys in the absence of inhibitor (P < 0.001). However, GSK-3β inhibition did not further decrease the number of apoptotic cells in empagliflozin-treated kidneys post I/R (P > 0.05 vs. EMPA) (Fig. 8A,B). Meanwhile, SB216763 effectively reduced the renal inflammatory response, to a level equivalent to that in EMPA-treated mice (Fig. 8C–E). We next evaluated the phosphorylation level of GSK-3β upon SB216763 administration. Notably, a similar effect on GSK-3β phosphorylation as that stimulated by empagliflozin was exerted by pharmacological inhibition of GSK-3β following renal I/R (P < 0.05 SB + I/R vs. I/R, Fig. 9). It is worth mentioning that GSK-3β inhibition did not enhance GSK-3β phosphorylation in empagliflozin-treated mice (P > 0.05 SB + EMPA vs. EMPA) (Fig. 9), nor did SB216763 increase renoprotection in empagliflozin-treated mice. Nevertheless, GSK-3β inhibitor-treated mice were less susceptible to renal I/R injury, very similar to what we observed in the EMPA group, indicating the beneficial role of GSK-3β phosphorylation in renoprotection post I/R. Moreover, in order to deeply understand the mechanism of GSK-3β inhibition, β-catenin (Fig. 10A) and Nrf2 (Fig. 10B), two downstream targets of GSK-3β signaling, were also examined in the current study. We found that mice treated with SB216763 or empagliflozin exhibited a higher expression levels of β-catenin and Nrf2 than those in the I/R group (P < 0.001).

## Discussion

To summarize, we found in our current study that empagliflozin exerts strong renoprotective effects against acute renal I/R injury, i.e., effectively preserves renal function and tubular morphology, inhibits cell apoptosis, suppresses inflammation and ameliorates acute renal damage. Furthermore, our data suggest that empagliflozin enhances GSK-3β phosphorylation post-I/R.

### SGLT2 inhibitors and renal protection

SGLT2 inhibitors (canagliflozin, dapagliflozin, empagliflozin, and ertugliflozin) are new hypoglycemic agents developed for the treatment of T2DM. The placebo-controlled randomized EMPA-REG OUTCOME trial^5^ recruited a total of 7020 patients with T2DM and established cardiovascular disease and revealed that patients treated with empagliflozin had lower rates of clinically relevant renal events or a lower risk of kidney disease progression than did those treated with placebo. A follow-up exploratory analysis of the EMPA-REG OUTCOME trial further evaluated the urinary albumin-to-creatinine ratio (UACR) and showed that empagliflozin may provide both short-term and long-term benefits with respect to urinary albumin excretion^22^. Later, by recruiting a total of 10,142 participants with T2DM with high cardiovascular risk, the CANVAS trial^6^ further supported the renoprotective role of an SGLT2 inhibitor (canagliflozin) in T2DM. Consistent with this, the CREDENCE trial recruited patients with T2DM and albuminuric chronic kidney disease (CKD) and revealed that those who received canagliflozin had a significantly lower risk of end-stage kidney disease or death from renal causes than did those receiving placebo^7^. Meanwhile, even in T2DM patients with CKD stages 3b and 4, dapagliflozin was shown to effectively reduce UACR and improve clinical outcomes^23^.

Strikingly, recent and accumulating evidence suggests that SGLT2 inhibitors also offer renoprotection or prevent kidney function from failing in patients without T2DM. The EMPEROR-Reduced trial revealed that empagliflozin is associated with a lower risk of serious renal outcomes in the presence or absence of diabetes^24^. Subsequently, the DAPA-CKD trial confirmed the beneficial renoprotective effects of dapagliflozin in patients with CKD, with or without T2DM^8^. Taken together, large clinical trials all showed that SGLT2 inhibitors exerted powerful renoprotective properties in nondiabetic individuals. Regarding renal I/R injury in the perioperative period, the most important question is whether SGLT2 inhibitors play a protective role against renal IR injury in nondiabetic individuals. It was previously shown that empagliflozin protects vital organs such as brains^25^ and hearts^11,26^ against I/R injury in a glucose-independent manner. Consistently, we also found that empagliflozin was renoprotective. Although there are no clinical data available, using a nondiabetic mouse model, we found that empagliflozin effectively improves renal function in the early post-I/R period following bilateral renal artery clamping. The results from our current preclinical study are in line with what has been observed by Chang et al., who showed that dapagliflozin pretreatment reduces acute renal damage post-I/R in mice in a glucose-independent manner^9^. Meanwhile, our findings are in accordance with findings reported by Ala et al*.* who found empagliflozin could enhance autophagy and mitochondrial biogenesis after renal Ischemia/reperfusion injury^27^. Our work is also in line with Zhang et al., who showed that luseogliflozin, an SGLT2 inhibitor, ameliorates renal fibrosis after prolonged renal I/R injury in nondiabetic mice^10^. Therefore, our current study may provide rationale for future clinical trial study designs.

### Empagliflozin inhibits apoptosis and the inflammatory response

It is well established that empagliflozin has cardio- or neuroprotective properties against I/R injury^25,26^. To the best of our knowledge, this current study is the first to elucidate the efficiency of empagliflozin in renal I/R injury. The underlying mechanism of empagliflozin-involved renoprotection has not been fully elucidated. The pathophysiological process of renal I/R often comprises multiple interactions of cell necrosis and apoptosis. B-cell lymphoma-2 (Bcl-2) is an anti-apoptotic protein in the Bcl-2 signaling pathway, which was first found in acute lymphoblastic leukemia^28^ and can inhibit programmed cell death^29^. Bcl-2-associated X protein (Bax) is a proapoptotic member of the Bcl-2 family, the activation of which in response to stress leads to cell apoptosis^30^. Thus, an enhanced Bcl-2/Bax ratio reflects the survival of cells after stimuli. In the present study, the expression of Bcl-2 and Bax in the kidney was measured by immunohistochemistry. We found that empagliflozin treatment increases Bcl-2 expression and decreases Bax expression, accompanied by an increased Bcl-2/Bax ratio when compared with I/R mice. Our results suggest that empagliflozin may exert its renoprotective effect by mediating renal apoptosis-related signaling pathways. Meanwhile, TNF-α and IL-6 are important inflammatory mediators during the pathophysiological process of renal I/R injury. It has been demonstrated that deleterious stimuli such as ischemia or reperfusion injury can increase the levels of TNF-α and IL-6 and thereafter facilitate neutrophil infiltration and induce renal damage. We found in our study that empagliflozin could decrease renal IL-6 and TNF-α expression, suggesting that empagliflozin could protect the kidney against renal I/R by mediating the inflammatory response.

### RISK/SAFE pathway in empagliflozin-mediated renoprotection

Currently, activation of the survivor activating factor enhancement (SAFE) pathway has been evaluated by many organ-protective strategies and has emerged as a promising target for the development of anti-I/R therapeutic approaches^31^. STAT3 and STAT5 are vital molecules in this pathway that can be phosphorylated upon protective treatment^32^. We previously demonstrated that phosphorylation of STAT3 and STAT5 are necessary steps in ischemic preconditioning in rat lungs^33^. Meanwhile, others also showed that remote preconditioning protected hearts against myocardial I/R injury via phosphorylation of STAT3^34^. In contrast, we did not find alterations in the phosphorylation levels of STAT3 and STAT5 after empagliflozin treatment when compared with I/R kidneys. This suggests that activation of the SAFE pathway does not contribute to the mechanism of empagliflozin-induced renoprotection.

The reperfusion injury salvage kinase (RISK) pathway, first proposed by Yellon’s group, is a major prosurvival signaling pathway associated with reperfusion injury^35,36^. We studied the key components in this pathway and found that the ratio of p-ERK1/2 to total ERK1/2 was not further elevated post-I/R in empagliflozin-treated kidneys, which was equivalent to the phosphorylation levels we observed in I/R mice, indicating that an alternative pathway may participate in empagliflozin-induced renoprotection. GSK-3β is a major downstream molecule of the RISK pathway. Phosphorylation of serine-9 in GSK-3β results in the suppression of GSK-3β activity^37^. Abundant studies have shown that protective strategies such as ischemic conditioning^14^ or volatile anesthetics^38^ could induce GSK-3β phosphorylation post-I/R, thereby promoting cell survival. Our current results confirmed these previous findings by demonstrating that empagliflozin enhanced the phosphorylation level of GSK-3β after I/R, suggesting that empagliflozin protects the kidney by targeting GSK-3β but not the ERK1/2 pathway. Additionally, we tested the effects of applying SB216763, a GSK-3β specific inhibitor, at the onset of renal reperfusion. We found that empagliflozin suppresses the activation of GSK-3β (via phosphorylation), and SB216763 mimics the effect of empagliflozin. Specifically, SB216763 effectively ameliorated renal damage, prevented histological alterations, and inhibited apoptosis after I/R. Similarly, we^14^ and others^39^ previously showed that SB216763 produces organ protection. Therefore, by extrapolation, we conclude that empagliflozin exerts its renoprotective effect similar to GSK-3β inhibition.

To further understand the molecular mechanism underlying GSK-3β inhibition and the protective role of empagliflozin, we examined several downstream targets of GSK-3β inhibition. GSK-3β plays a key role in regulation of the Wnt/β-catenin signaling pathway. The inhibition of GSK-3β activity results in an increase in β-catenin level^40^. It has been reported that protective stimuli such as SB216763^16^ or ischemic preconditioning^41^ could enhance GSK-3β phosphorylation and β-catenin accumulation during hepatic I/R. In agreement with these previous findings, we showed that SB216763 exerted protective effects against renal injury induced by I/R via activation of the β-catenin signaling pathway. Furthermore, we also found that empagliflozin activated the β-catenin pathway, mimicking the effect of GSK-3β inhibition. Next, we examined the expression levels of nuclear factor erythroid 2-related factor 2 (Nrf2), a downstream regulator of GSK-3β. Recent studies have shown that inhibiting GSK-3β could regulate Nrf2^42^. Meanwhile, using the Nrf2 knockout animal model, Kay et al. showed that sauchinone produced hepatic protection via GSK-3β inhibition, which further led to the activation of Nrf2^43^. Consistent with these findings, we found that empagliflozin, similar to SB216763, ameliorated renal damage post-I/R by activating the Nrf2 signaling pathway. Taken together, empagliflozin was shown to inhibit the activation of GSK-3β (by phosphorylation) and could activate GSK-3β downstream targets, suggesting that empagliflozin exerts its renoprotective effects via GSK-3β inhibition.

## Limitations and conclusions

We acknowledge several limitations of this study. First, we only conducted a short-term observation, i.e., 45 min of renal ischemia followed by 24 h of reperfusion; whether empagliflozin offers long-term protection after renal I/R injury requires further determination. Furthermore, renal I/R injury can cause acute kidney failure, which leads to high mortality rates and rapid kidney dysfunction; however, we did not document the mortality rate during our experiment, thus, it is unknown whether empagliflozin would improve survival post-I/R. Second, empagliflozin was administered intragastrically to the mice for 2 days (1 mg/kg body weight); however, the clinical dose for humans is 10–25 mg/60 kg/day. Although empagliflozin was shown to have renoprotective effects against renal I/R injury in our study, it is worth exploring its renal protective efficiency within the clinical dose range in future studies. Third, only the SAFE and RISK signaling pathways were examined in our study; other cell survival-associated signaling pathways may possibly contribute to empagliflozin-induced renoprotection. The current experimental design did not link GSK-3β inhibitors to proinflammatory markers assessed in the current study; thus, it remains unknown whether GSK inhibition alters the inflammatory response post renal I/R. Fourth, empagliflozin postconditioning (drug given after I/R) has more therapeutic advantages versus preconditioning. We only evaluated the prophylactic effects of SGLT2 inhibition against renal I/R. Therefore, postconditioning-induced renal protection warrants further investigation.

In summary, empagliflozin pretreatment has strong renoprotective effect in a nondiabetic mouse model of renal I/R injury, as seen by a reduction in renal damage and preservation of renal function. The protective action of empagliflozin is similar to that of GSK-3β inhibition. The present study may provide novel therapeutic approaches for the treatment of renal I/R injury in the postoperative period (Supplementary Information).

## Supplementary Information

Supplementary Information.

## Data Availability

The datasets used and/or analyzed during the current study are available from the corresponding author on reasonable request.
