# Supplementary material for: Empagliflozin protects against renal ischemia/reperfusion injury in mice
Source: Sci Rep. 2022 Nov 11;12:19323. doi: 10.1038/s41598-022-24103-x (PMC9652474; doi:10.1038/s41598-022-24103-x)
Supplement: Supplementary file 1 — Supplementary Information. [file 41598_2022_24103_MOESM1_ESM.pdf]

The phosphorylation levels of STAT3, STAT-5, ERK1/2 and GSK-3β were normalized to their respective total protein. We first cut the blots prior to hybridization with antibodies according to their molecular weight. Then, we stripped the bands and re-probed the membrane with total protein antibodies at the same molecular weight in the same membrane.
For detecting IL-6, and TNF-α, we cropped the membrane into small strips before hybridization with antibodies according to the protein molecular weight.
We have provided all replicates as follows.

Figure 3B:

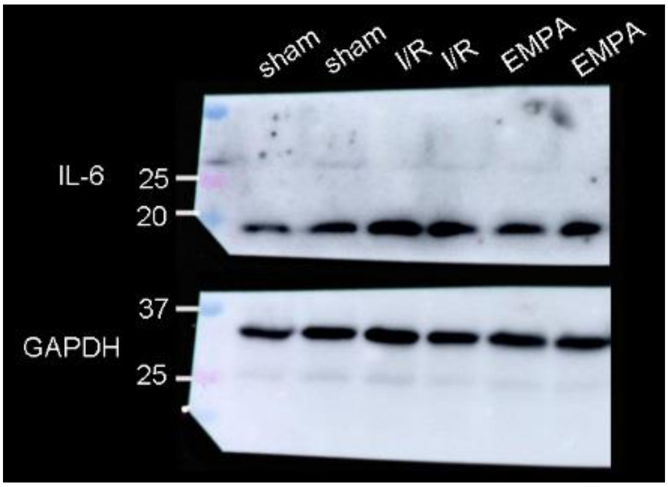

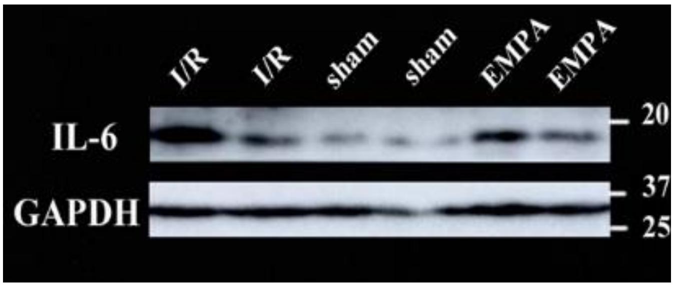

Figure 3D:

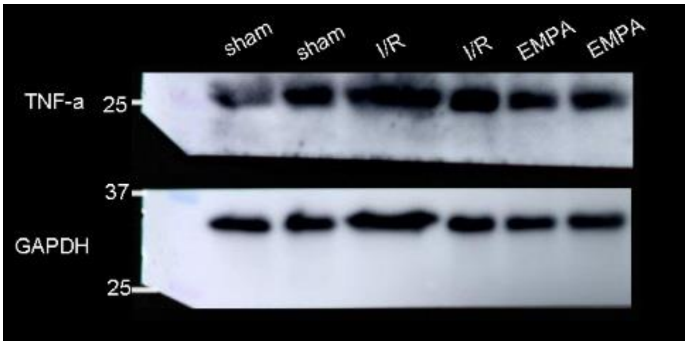

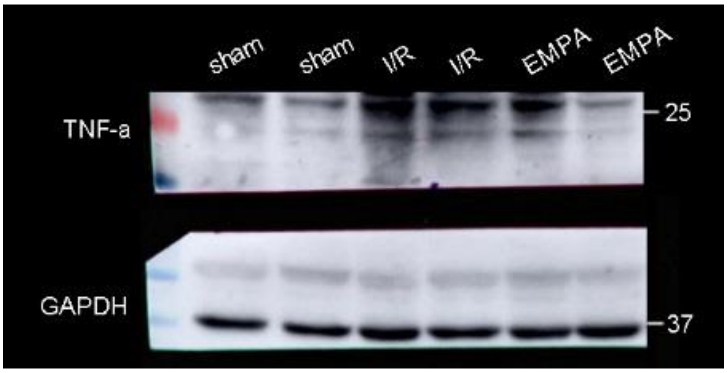

Figure 6A:

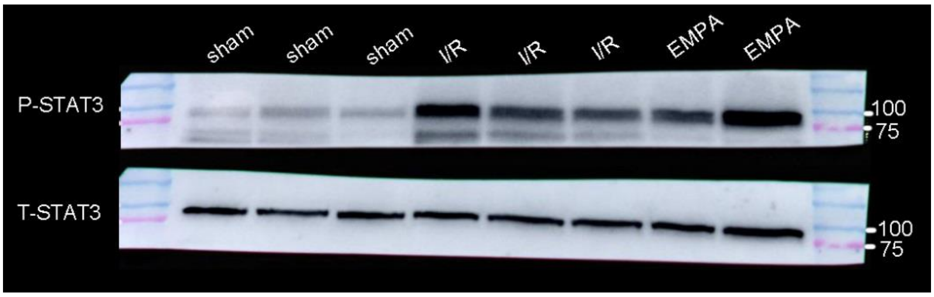

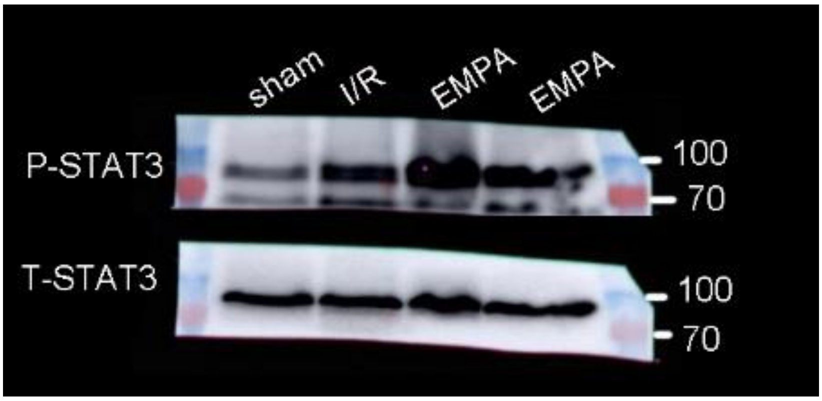

Figure 6B:

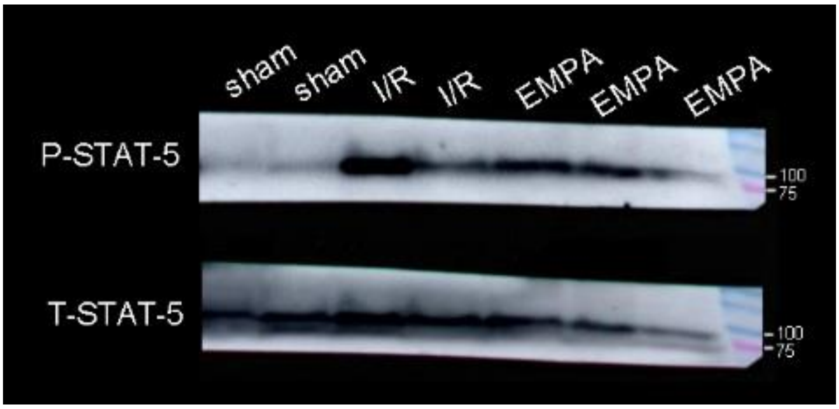

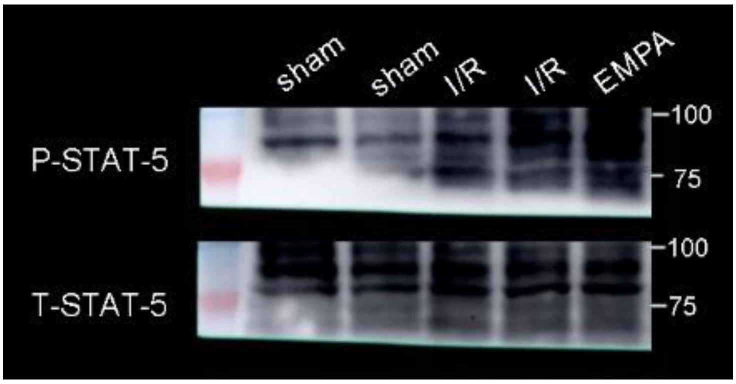
sham
sham
I/R
I/R
EMPA
P-STAT-5
100
75
T-STAT-5
100
75

Figure 6C:

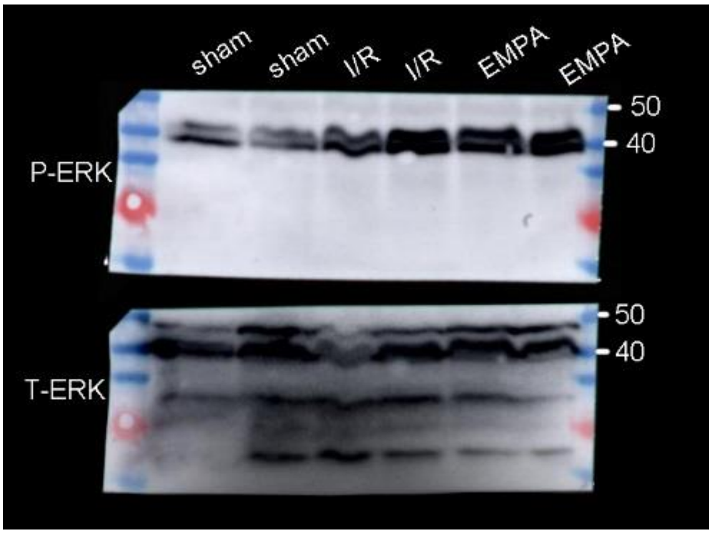
sham
sham
I/R
I/R
EMPA
EMPA
P-ERK
50
40
T-ERK
50
40

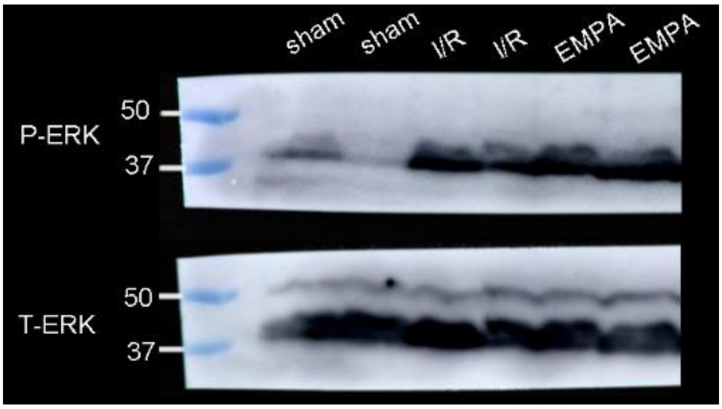
sham
sham
I/R
I/R
EMPA
EMPA
P-ERK
50
37
T-ERK
50
37

Figure 6D:

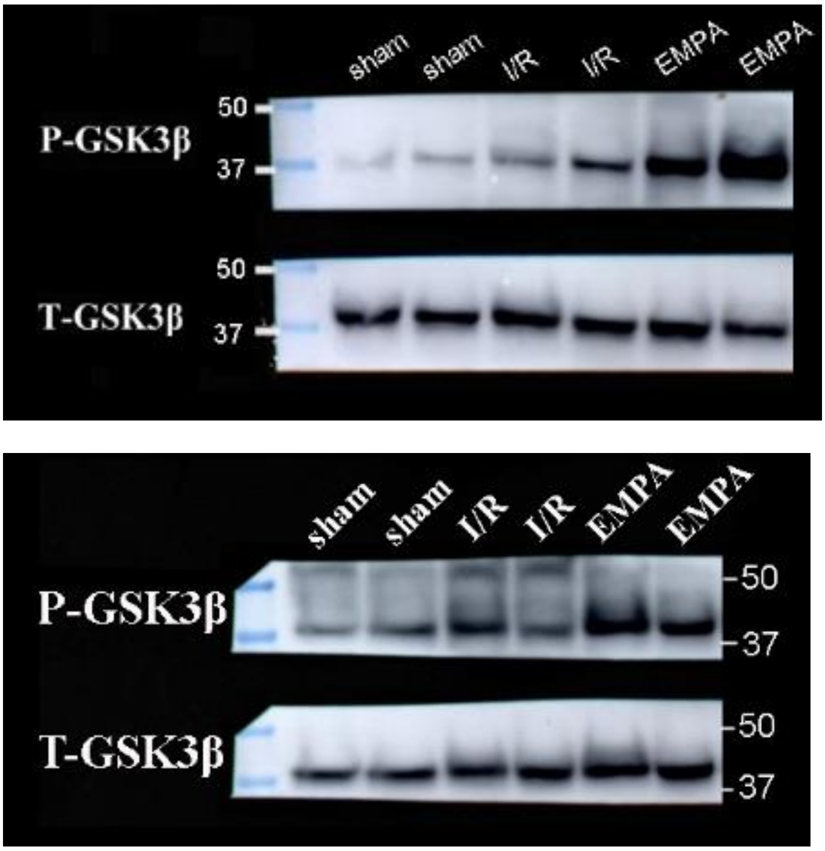

Figure 9:

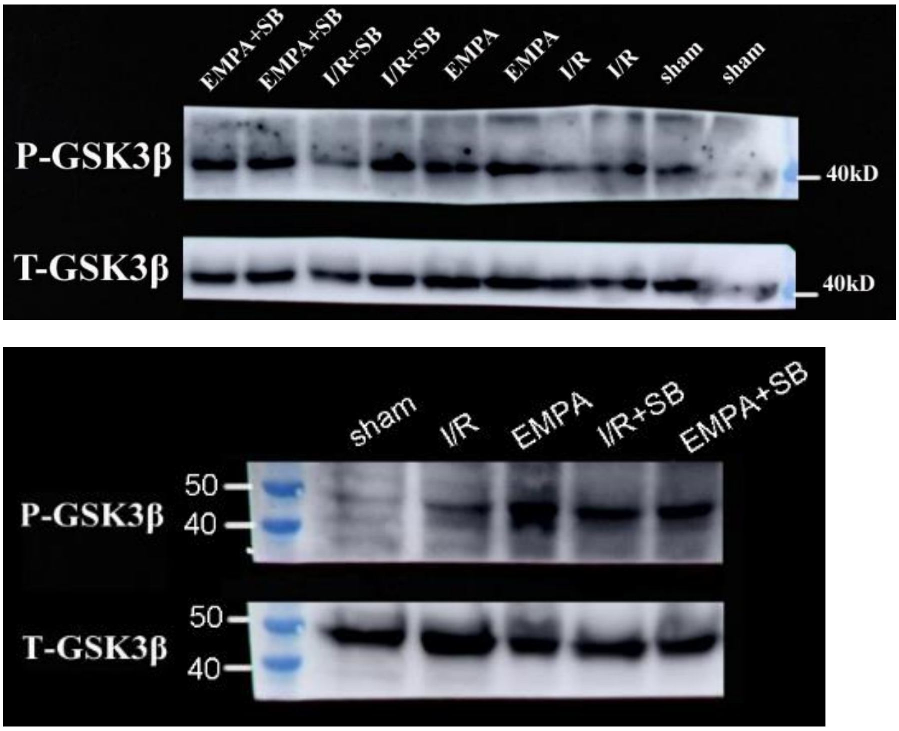

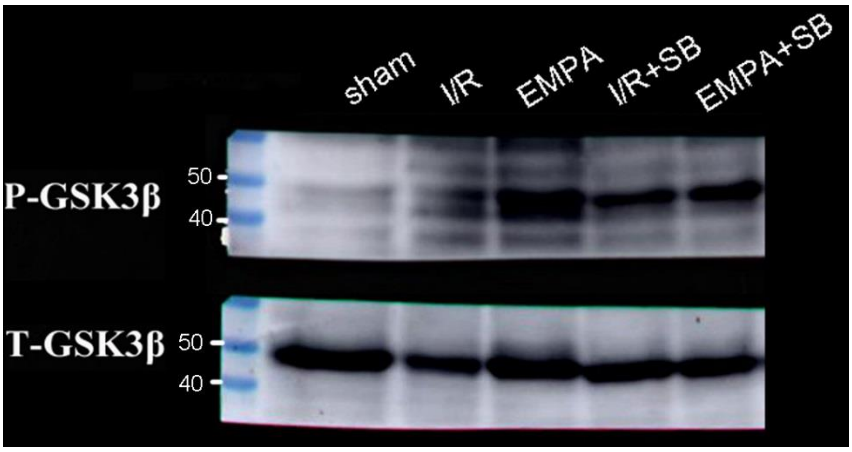
sham
I/R
EMPA
I/R+SB
EMPA+SB
P-GSK3β
50
40
T-GSK3β
50
40
